# Long‐term metformin use for Alzheimer's disease prevention?

**DOI:** 10.1002/alz.70147

**Published:** 2025-04-12

**Authors:** Timothy Daly, Bruno P. Imbimbo

**Affiliations:** ^1^ UMR 1219 Bordeaux Population Health Université de Bordeaux & INSERM Bordeaux France; ^2^ UMR 5164, ImmunoConcept University of Bordeaux & CNRS Bordeaux France; ^3^ Bioethics Program FLACSO Argentina Buenos Aires Argentina; ^4^ Research & Development Chiesi Farmaceutici Parma Italy

1

In *Alzheimer's & Dementia*, Zheng and colleagues published results from a UK‐based cohort study of 210,237 participants, suggesting that metformin use reduced dementia risk beyond the level observed in patients with milder diabetes and better health profiles.^1^

We build on the authors’ observation that longer metformin treatment duration was associated with greater dementia risk reduction, along with evidence from epidemiology, the pathological mechanisms of Alzheimer's disease (AD), and metformin's availability, to propose that long‐term metformin at 500 mg/day in high‐risk individuals could serve as a preventive strategy to reduce the risk or delay the onset of AD and related dementias.

This approach is conceptually analogous to the well‐established use of statins in cardiovascular medicine, where long‐term administration in at‐risk populations has been shown to reduce the incidence of major cardiovascular events.^2^ We propose that long‐term metformin use could be investigated as a repurposed drug for AD prevention.

Type 2 diabetes mellitus (T2DM) has shown a strong metabolic and inflammatory association with dementia.^3^ The study by Zheng et al.^1^ is among a growing number of epidemiological studies reporting a protective effect of metformin on dementia risk, consistently indicating that longer metformin treatment duration is associated with greater risk reduction.^4^, ^5^, ^6^ Beyond epidemiological evidence, mechanistic insights also support the notion that metformin may exert neuroprotective effects through multiple pathways. These include not only its metabolic effects^3^ but also its role in attenuating general mechanisms of cellular aging,^7^ slowing brain aging,^8^ and modulating key AD‐related pathologies, including amyloid and tau.^9^ Furthermore, metformin at 500 mg/day is the most widely prescribed antidiabetic drug worldwide, is recognized as an essential medicine by the World Health Organization as of 2023, and is available off‐patent at low cost in most countries.^10^

Just as statins are prescribed to individuals with hyperlipidemia or other cardiovascular risk factors before the onset of overt disease,^2^ metformin at 500 mg/day could be administered to individuals in the preclinical or prodromal stages of AD, such as older adults with mild cognitive impairment (MCI), insulin resistance, and/or a strong genetic predisposition (e.g., apolipoprotein E (*APOE*) ε4 homozygotes, pathogenic Sortilin‐related Receptor 1 (*SORL1)* genetic variants, or individuals with a maternal history of AD). By targeting key pathways involved in AD pathogenesis, metformin could potentially modify disease progression, making it a promising candidate for repurposing in the prevention of AD.

In conclusion, metformin is a safe, affordable, and widely used anti‐T2DM drug, with growing evidence suggesting that its long‐term use is associated with a lower risk of cognitive decline. An ongoing 18‐month, double‐blind, placebo‐controlled trial—the Metformin in Alzheimer's Dementia Prevention study (NCT04098666)—is investigating the protective effects of doses up to 2000 mg/day, with results expected in 2027. Although this is an important study, it will not directly assess the therapeutic value of long‐term use, which we consider the strongest argument for metformin in dementia risk reduction. Therefore, we advocate for a controlled, long‐term intervention in high‐risk individuals to provide high‐level evidence on metformin's role in the prevention of AD and related disorders.

## CONFLICT OF INTEREST STATEMENT

Dr. Timothy Daly has no conflicts of interest to declare. Dr. Bruno P. Imbimbo is an employee at Chiesi Farmaceutici. He is listed as an inventor in a number of Chiesi Farmaceutici's patents of anti‐Alzheimer's drugs.

Author disclosures are available in the Supporting Information.

## FUNDING INFORMATION

Timothy Daly is a postdoctoral research fellow funded by the “Institut National de la Santé et de la Recherche Médicale” (INSERM) on the MEMENTO project (France).

## Supporting information



Supporting Information
