# Supplementary material for: Long‐term metformin use for Alzheimer's disease prevention?
Source: Alzheimers Dement. 2025 Apr 12;21(4):e70147. doi: 10.1002/alz.70147 (PMC11992593; doi:10.1002/alz.70147)
Supplement: Supplementary file 1 — Supporting Information [file ALZ-21-e70147-s001.pdf]

ICMJE DISCLOSURE FORM

**Date:**	8th February 2025
**Your Name:**	Timothy Daly
**Manuscript Title:**	"Long-Term Metformin Use for Alzheimer's Disease Prevention?
**Manuscript Number (if known):**	ADJ-D-25-00339,

In the interest of transparency, we ask you to disclose all relationships/activities/interests listed below that are related to the content of your manuscript. "Related" means any relation with for-profit or not-for-profit third parties whose interests may be affected by the content of the manuscript. Disclosure represents a commitment to transparency and does not necessarily indicate a bias. If you are in doubt about whether to list a relationship/activity/interest, it is preferable that you do so.

The author's relationships/activities/interests should be defined broadly. For example, if your manuscript pertains to the epidemiology of hypertension, you should declare all relationships with manufacturers of antihypertensive medication, even if that medication is not mentioned in the manuscript.

In item #1 below, report all support for the work reported in this manuscript without time limit. For all other items, the time frame for disclosure is the past 36 months.

		**Name all entities with whom you have this relationship or indicate none (add rows as needed)**	**Specifications/Comments (e.g., if payments were made to you or to your institution)**
		**Time frame: Since the initial planning of the work**	
**1**	All support for the present manuscript (e.g., funding, provision of study materials, medical writing, article processing charges, etc.) **No time limit for this item.**	X **None**	
			Click the tab key to add additional rows.
		**Time frame: past 36 months**	
**2**	Grants or contracts from any entity (if not indicated in item #1 above).	☐ **None**	
		INSERM France 2024—2025	Paid to me
		FLACSO Argentina 2021—2025	Paid to me
**3**	Royalties or licenses	X **None**	

		Name all entities with whom you have this relationship or indicate none (add rows as needed)	Specifications/Comments (e.g., if payments were made to you or to your institution)
**4**	Consulting fees	X **None**	
**5**	Payment or honoraria for lectures, presentations, speakers bureaus, manuscript writing or educational events	X **None**	
**6**	Payment for expert testimony	X **None**	
**7**	Support for attending meetings and/or travel	☐ **None**	
		INSERM 2024	Paid to me
		FLACSO Argentina 2024	Paid to me
		FLACSO Argentina 2023	Paid to me
**8**	Patents planned, issued or pending	X **None**	
**9**	Participation on a Data Safety Monitoring Board or Advisory Board	X **None**	

		Name all entities with whom you have this relationship or indicate none (add rows as needed)	Specifications/Comments (e.g., if payments were made to you or to your institution)
**10**	Leadership or fiduciary role in other board, society, committee or advocacy group, paid or unpaid	X **None**	
**11**	Stock or stock options	X **None**	
**12**	Receipt of equipment, materials, drugs, medical writing, gifts or other services	X **None**	
**13**	Other financial or non-financial interests	X **None**	

Please place an "X" next to the following statement to indicate your agreement:

X I certify that I have answered every question and have not altered the wording of any of the questions on this form.

ICMJE DISCLOSURE FORM

Date: 2/8/2025

Your Name: Bruno Pietro Imbimbo

Manuscript Title: Long-Term Metformin Use for Alzheimer's Disease Prevention?

Manuscript Number (if known): ADJ-D-25-00339

In the interest of transparency, we ask you to disclose all relationships/activities/interests listed below that are related to the content of your manuscript. "Related" means any relation with for-profit or not-for-profit third parties whose interests may be affected by the content of the manuscript. Disclosure represents a commitment to transparency and does not necessarily indicate a bias. If you are in doubt about whether to list a relationship/activity/interest, it is preferable that you do so.

The author's relationships/activities/interests should be defined broadly. For example, if your manuscript pertains to the epidemiology of hypertension, you should declare all relationships with manufacturers of antihypertensive medication, even if that medication is not mentioned in the manuscript.

In item #1 below, report all support for the work reported in this manuscript without time limit. For all other items, the time frame for disclosure is the past 36 months.

		Name all entities with whom you have this relationship or indicate none (add rows as needed)	Specifications/Comments (e.g., if payments were made to you or to your institution)
**Time frame: Since the initial planning of the work**			
1	All support for the present manuscript (e.g., funding, provision of study materials, medical writing, article processing charges, etc.) **No time limit for this item.**	☐ **None**	
		Bruno P. Imbimbo is an employee at Chiesi Farmaceutici	
			Click the tab key to add additional rows.
**Time frame: past 36 months**			
2	Grants or contracts from any entity (if not indicated in item #1 above).	☒ **None**	
3	Royalties or licenses	☒ **None**	

		Name all entities with whom you have this relationship or indicate none (add rows as needed)	Specifications/Comments (e.g., if payments were made to you or to your institution)
**4**	Consulting fees	☒ **None**	
**5**	Payment or honoraria for lectures, presentations, speakers bureaus, manuscript writing or educational events	☒ **None**	
**6**	Payment for expert testimony	☒ **None**	
**7**	Support for attending meetings and/or travel	☒ **None**	
**8**	Patents planned, issued or pending	☐ **None**	
		Bruno P. Imbimbo is listed as an inventor in a number of Chiesi Farmaceutici's patents of anti-Alzheimer drugs.	
**9**	Participation on a Data Safety Monitoring Board or Advisory Board	☒ **None**	

		Name all entities with whom you have this relationship or indicate none (add rows as needed)	Specifications/Comments (e.g., if payments were made to you or to your institution)
**10**	Leadership or fiduciary role in other board, society, committee or advocacy group, paid or unpaid	☒ **None**	
**11**	Stock or stock options	☒ **None**	
**12**	Receipt of equipment, materials, drugs, medical writing, gifts or other services	☒ **None**	
**13**	Other financial or non-financial interests	☒ **None**	

Please place an "X" next to the following statement to indicate your agreement:

☒ I certify that I have answered every question and have not altered the wording of any of the questions on this form.
